# Efficiency of primary care in Brazilian capitals and management models: 2008–2019

**DOI:** 10.11606/s1518-8787.2025059006346

**Published:** 2025-05-02

**Authors:** André Luis Paes Ramos, Marismary Horsth De Seta, Marcelo Battesini

**Affiliations:** I Fundação Oswaldo Cruz. Escola Nacional de Saúde Pública Sergio Arouca. Programa de Pós-graduação em Saúde Pública. Rio de Janeiro, RJ, Brasil; II Universidade Federal de Santa Maria. Programa de Pós-Graduação em Gestão de Organizações Públicas. Santa Maria, RS, Brasil

**Keywords:** Primary Health Care, Outsourced Services, Health Evaluation, Efficiency

## Abstract

**OBJECTIVE:**

To analyze efficiency in primary health care in Brazilian state capitals in the period 2008–2019, considering the management model: direct public administration or administration assigned to third parties.

**METHODS:**

This is an evaluative study with an analytical objective, using publicly available secondary data, analyzed cross-sectionally (2019) and longitudinally (2008, 2012, 2016, and 2019). Demographic and socioeconomic data and seven indicators related to primary health care were used to characterize the cities and their primary health care (primary care coverage, hospitalizations for primary care-sensitive conditions, tuberculosis cures, infant, maternal, and premature mortality due to chronic conditions, incidence of congenital syphilis). To these indicators were added health and primary health care expenditures and data envelopment analysis focused on outputs (without admitting a reduction in expenditures) to calculate efficiency in 2008, 2012, 2016, and 2019. The Malmquist index was used to identify possible productivity gains between 2008 and 2019.

**RESULTS:**

Four capitals identified as being managed by third parties (São Paulo, Rio de Janeiro, Porto Alegre, and Fortaleza) did not achieve greater efficiency compared to direct public administration, nor did they evolve when comparing their own results over time. In 2019, and in the longitudinal approach, only capitals managed by direct public administration obtained the maximum relative efficiency index in the comparison between capitals. Twelve capitals with direct public administration remained efficient in all years, while those with third-party administration consistently showed weak inefficiency in primary health care, i.e. in the period studied, the relationship between investments in primary health care and results obtained is lower than that achieved by the efficient capitals.

**CONCLUSIONS:**

We found no evidence of efficiency gains with the adoption of third-party management. It should be noted that productive efficiency does not necessarily mean that health needs are met.

## INTRODUCTION

Countries with universal health systems invest heavily in Primary Health Care (PHC) to access the system and solve the most common needs.^1,2^

In Brazil, municipalities are mainly responsible for managing and implementing PHC policies. State capitals stand out due to their structural, economic, political, and social conditions, and because they are regional hubs of reference, not just for the surrounding municipalities.

There are various projects in dispute about the best way to manage and provide health services, and the debate about the greater or lesser presence of the state in the provision of social policies. One argument is the supposed greater efficiency of the private sector over the state sector^3^. Brazilian public managers point to tendering laws and the Fiscal Responsibility Law (LRF) as constraints on exclusive public management, making it necessary to use alternative, more flexible management models for purchasing and hiring staff.^4^

Alternatives to exclusive public administration are proliferating: state foundations under public or private law, public companies, non-governmental organizations, social organizations (OS), Public Law Civil Society Organizations, Civil Society Organizations, Autonomous Social Services. Mixed arrangements are frequent, in which part of the health units are managed by the direct or indirect public administration, and another part is outsourced to private institutions; different models can coexist in the same health unit.

Created in 1998, the OSs represent the main strategy for outsourcing the administration of public health units, but their expansion has not been accompanied by studies on whether or not the promised efficiency has been achieved, especially in PHC. Studies on the performance of hospital care managed by OS or public administration compare hospitals by indicators of each establishment, without focusing on results and impacts on the health of the population assisted in the system in which they are inserted.

Recent work^5,6^ has analyzed PHC performance - understood as the extent to which the health system in Brazilian capitals achieves the proposed objectives^7^. However, efficiency was not analyzed and the expenses incurred to achieve the results were not considered.

Efficiency, a constitutional principle for public administration^8^, is listed in the Health Sciences Descriptors (DeCs) as the ratio between effort and resources employed. The efficient relationship between the results obtained and the quantity of inputs used (production function) is not easily determined in public health and requires the analysis of best practices from virtuous references (benchmarks), which delimit a limiting frontier for units that share similar conditions^9^. However, the use of efficiency measures should not be used to reduce the allocation of public resources, given their minor contribution to the composition of health spending in Brazil, which is predominantly private.^9^

This study aims to analyze the efficiency of PHC in Brazilian state capitals in the period 2008–2019, based on two questions. Firstly, whether the capitals are efficient in providing PHC; and secondly, whether the PHC management model adopted has an impact on efficiency.

## METHODS

This is an evaluative study with an analytical objective, using publicly available secondary data, analyzed cross-sectionally (2019) and longitudinally (2008, 2012, 2016, and 2019).

The study was carried out in two phases. In the first, PHC in the 27 state capitals was characterized using the usual indicators^5,10,11^ (Table 1) referring to population data, management model, public spending and collection, and the percentage of the population covered by supplementary health plans (population in SH), prioritizing the most recent information (between 2020 and 2021). *Per capita* values were estimated to reduce the influence of socio-economic disparities and population size. Two of these indicators were also used in the second phase (health expenditure and PHC expenditure).

**Table 1:** Characterization of the capitals according to selected indicators (2018, 2020 and 2021).

Capital/UF	Population^b^	PHC units under TA^a^	GDP (R$/hab)^b^	Health expenditure (R$/inhabitant)^c^	PHC expenditure (R$)^c^	% FPM^c^	% Own resources in Health^c^	% SS population^d^	% Personal Expenses LRF^c^
	2020	2021	2018	2020	2020	2020	2020	(Dec 2020)	2020
Porto Velho (RO)	539,354	0	32,042.66	706.62	52.12	17.68	23.1	18.2	50.79
Rio Branco (AC)	413,418	0	22,287.70	319.46	267.98	35.50	16.1	11.2	41.92
Manaus (AM)	2,219,580	0	36,445.75	503.11	163.07	10.83	23.6	27.6	40.86
Boa Vista (RR)	419,652	0	26,752.67	593.62	154.42	32.83	19.3	9.8	43.14
Belém (PA)	1,499,641	0	21,191.47	514.26	78.92	15.52	27.1	28.6	45.25
Macapá (AP)	512,902	0	22,181.72	438.6	206.17	33.66	17.5	12.8	50.35
Palmas (TO)	306,296	0	32,293.89	841.52	264.63	20.50	21.1	21.9	48.46
São Luís (MA)	1,108,975	0	30,699.57	819.87	58.49	17.42	21.6	29.0	44.78
Teresina (PI)	868,075	0	24,333.00	1,677.84	127.39	19.56	42.2	30.8	47.17
Fortaleza (CE)	2,686,612	20	25,356.73	958.16	172.42	12.84	28.7	36.4	46.03
Natal RN)	890,480	0	27,122.37	836.78	41.25	13.55	30.7	37.1	50.62
João Pessoa (PB)	817,511	0	25,035.80	880.55	150.46	14.12	21.1	31.9	47.58
Recife (PE)	1,653,461	0	31,994.38	936.57	390.40	10.58	23.2	39.9	46.99
Maceió (AL)	1,025,360	0	22,126.34	765.92	59.01	16.62	21.3	29.2	43.72
Aracaju (SE)	664,908	0	26,622.38	757.37	202.57	17.06	22.3	39.5	46.46
Salvador (BA)	2,886,698	1	22,232.68	635.05	30.04	12.66	22.2	29.7	36.93
Belo Horizonte (MG)	2,521,564	0	36,759.66	1,463.70	317.00	4.86	22.9	48.7	41.85
Vitória (ES)	365,855	0	73,632.55	756.80	308.54	11.73	18.3	64.2	46.31
Rio de Janeiro (RJ)	6,747,815	217	54,426.08	630.30	132.45	1.56	16.8	47.5	56.24
São Paulo (SP)	12,325,232	74	58,691.90	1,042.83	403.64	0.54	21.8	50.5	33.58
Curitiba (PR)	1,948,626	0	45,458.29	1,030.94	350.04	4.57	20.1	55.3	42.25
Florianópolis (SC)	508,826	0	42,719.16	705.89	517.22	7.61	17.6	42.0	48.68
Porto Alegre (RS)	1,488,252	109	52,149.66	1,180.80	196.45	4.27	18.1	43.6	41.81
Campo Grande (MS)	906,092	0	32,942.46	1,535.96	263.26	4.35	25.4	30.5	50.39
Cuiabá (MT)	617,848	0	39,043.32	1,597.80	101.29	5.88	34.7	40.7	45.63
Goiânia (GO)	1,536,097	0	33,004.01	1,037.47	55.66	7.04	19.9	38.9	44.12
Brasília (DF)	3,055,149	0	85,661.39	1,137.83	47.70	0.74	17.75	34.3	42.05

UF: Federated Unit - population used by the Federal Court of Auditors; PHC: primary health care - establishments under third-party administration; GDP: gross domestic product; FPM: municipal participation fund in total revenues; SS: supplementary health - coverage by plans; LRF: Fiscal Responsibility Law.

Sources: ^a^National Registry of Health Establishments (CNES); ^b^Brazilian Institute of Geography and Statistics (IBGE); ^c^Information System on Public Health Budgets - liquidated expenses; ^d^National Supplementary Health Agency (ANS).

Considering that the quality of the administration of health services can have a positive impact on their performance^9^, the predominant PHC management models in each capital were identified based on information obtained from local managers^5^, with a bibliographic search for updates. This procedure resulted in the following categories: exclusive direct public administration (DA) and administration granted to third parties (TA). The indicators were analyzed using descriptive statistics and Pearson’s correlation.

In the second phase, the efficiency of PHC management in the capital cities was studied using data envelopment analysis (DEA), a non-parametric multidimensional method used to estimate the efficiency of decision making units (DMUs), based on mathematical linear programming^12^. In addition to spending per capita on health and PHC, health indicators from the 2013–2015 Guidelines and Goals Pact (PDM)^13^ (Chart) were used as variables, calculated from national data. The DEA models were analyzed and solved in the R-Studio environment (R Core Team, 2022), using the benchmarking package and its functions.

**Chart:** Inputs (Xi) and Outputs (Yi) used in the application of data envelopment analysis (DIA).

Indicator	Description	Source	Variable
Spending per capita on the Health Function	Number of resources allocated to the Health macro function	SIOPS/IBGE	X_1_
Spending per capita on Primary Care	Number of resources allocated to the Primary Care subfunction	SIOPS/IBGE	X_2_
Coverage of BC teams	PHC’s human resources, expressing the potential supply of basic actions and services and access to them	E-gestor AB	Y_1_
Proportion of ICSAB	ICSAB, expressing the risk of unnecessary hospitalization that can be reduced by effective PHC actions	SIH	Y_2_
Infant mortality rate	It is one of the main public health indicators. It reveals maternal and child health conditions and expresses the health situation of a community and inequalities between social groups and regions.	SIM / SINASC	Y_3_
Proportion of new cases of pulmonary tuberculosis cured	Indicates success in tuberculosis treatment, difficulties in monitoring identified cases and reduction in transmission	Sinan	Y_4_
CNCD premature mortality rate	Monitors the impact of public policies on the prevention and control of the main CNCDs (circulatory system, cancer, diabetes, and chronic respiratory diseases) and their risk factors in people under the age of 70	SIM / IBGE	Y_5_
Maternal mortality rate	Expresses the deaths of women of childbearing age from causes linked to pregnancy, childbirth and the puerperium, which are generally preventable and avoidable.	SIM / SINASC	Y_6_
Congenital syphilis incidence rate	Indicates the quality of prenatal care, since syphilis can be diagnosed and treated during pregnancy	Sinan	Y_7_

PHC: primary health care; BC: basic care; ICSAB: hospitalizations for primary care sensitive conditions; SIOPS: Public Health Budget Information System; IBGE: Brazilian Institute of Geography and Statistics; E-gestor AB: Primary Care Information and Management; SIH: Hospital Information System; SIM: Mortality Information System; SINASC: Live Birth Information System; Sinan: Notifiable Diseases Information System; CNCD: chronic non-communicable diseases.

DEA is based on the productivity of the DMUs analyzed, estimated by the ratio between the results obtained and the resources used. The efficiency of a DMU is obtained by solving the linear programming problem that optimizes the ratio $\theta =\text{ virtual output/virtual input }=\left[vy_{11}+v_{22}+\ldots +v_{\mathrm{nn}}\right]/\left[{\mathrm{ux}}_{11}+{\mathrm{ux}}_{22}+\ldots +{\mathrm{ux}}_{\mathrm{mm}}\right]$ , with θ ≥ 1, where v_i_ and u_i_ are the optimal weights (v_i_ and u_i_ ) obtained, with n output variables and m input variables^14^, the as a result of comparing the productivity of a DMU in relation to the maximum productivity defined by a latent frontier.

In general, DEA minimizes inputs and maximizes outputs*,* seeking greater efficiency or better performance^15^, and the efficient DMUs form a frontier that serves as a reference for the inefficient ones. Efficiency results from comparing the different abilities to convert inputs into outputs (productivity) and the endogenous efficiency frontier is identified by a mathematical linear programming model^16^. The steps used are: define DMU; select variables; define model; obtain and present results^12^. In addition, we estimate the change in productivity over time.

The 27 capitals were taken as DMUs because they have similar supply conditions and are local references in PHC. The input variables (X_i_, resources), public spending per inhabitant on the health function (X_1_) and on Primary Care (X_2_), understood in this study as synonymous with PHC, express investment in health and the degree of priority given by management to the first level of care. The variables used as output (Yi) included indicators of production (intermediate products) and effectiveness and outcomes (outcomes)^9^, as shown in the Chart.

The output variables Y_2_, Y_3_, Y_5_, Y_6,_ and Y_7_ (smallest-is-best) were taken in their inverse in the DEA analyses to assume the same direction as the others (largest-is-best) in the optimization.

The output-oriented variable return DEA model (VRS-O DEA) was used, referring to the way in which the efficiency frontier is defined and the orientation refers to the direction chosen to estimate the efficiency of inefficient DMUs^16^. Two mathematical models are used in the DEA VRS-O, represented in Equations 1 and 2^12^. The first presents the primal in the form of the envelope (definition of the frontier) and the second the dual in the form of the multipliers expressed algebraically (definition of the weights).


$$\max\theta_{0}|\text{ subject to: }X\lambda \leq x_{0};\theta y_{0}-Y\lambda \leq 0;e\lambda =1;\lambda \geq 0$$ Equation 1

in which, q_0_ is the efficiency of the DMU under analysis; X= {x_i_ } is a set of input variables; Y= {y_j_ } is a set of output variables; x_0_ and y_0_ are the input and output variables of the DMU under analysis; l is the vector of weights of the DMU under analysis; e is a vector with elements equal to 1; and l = 1 imposes the convexity condition.


$$\min\left[\left({\mathrm{vx}}_{0}-v_{0}\right)/{\mathrm{uy}}_{0}\right]|\text{ subject to: }\left[\left({\mathrm{vx}}_{i}-v_{0}\right)/{\mathrm{uy}}_{j}\right]\geq 1$$ Equation 2

in which x_i_ and y_j_ are *input* and *output* variables; and v_i_ and u_i_ the optimal weights.

The VRS-O model makes it possible to estimate the technical efficiencies (E_technical_) of the DMUs, based on a convex envelope defined by sections, seeking to maximize output levels considering the current consumption of inputs^12^. The output orientation is compatible with research in the public health area, whose budget is limited^9,17^ and is not intended to be reduced, even though a progressive improvement in PHC indicators is expected.

The E_technical_ scores estimated by the VRS DEA model express a component of global efficiency (E_global_), which also includes scale efficiency E_scale_ = E_technical_ / E_global_^12^. Thus, to identify whether a DMU operates at its most productive scale (economy of scale), it is necessary to estimate the DEA CRS model.^14^

The output-oriented variable return DEA model (VRS-O DEA) was estimated for the years 2019, 2016, 2012, and 2008 (cross-sectional analyses). In addition, E_global_ was estimated using the constant return DEA model (VRS-O DEA) to identify the E_scale_ of DMUs in 2019.

Cross-sectional analyses generate specific results on the relative efficiency of DMUs, without considering the fact that the phenomenon under investigation may evolve or decline over time. To observe these changes, the Malmquist index (MI) was estimated between 2008 and 2019 (longitudinal analysis). The MI is a global measure of DMU change over time (progress or regression) and expresses productivity gains between times t and t+1^15^ . MI is defined as the product of its components (MI = E_MI_ *T_MI_), which express the change in Efficiency (E_MI_, catch-up effect) and the change in Technology (T_MI_, frontier-shift effect), and is calculated by Equation 3.^16^


$$\mathrm{per}IM^{t+1}\left(x^{t+1},y^{t+1},x^{t},y^{t}\right)=\left[\frac{D^{t+1}\left(x^{t+1},y^{t+1}\right)}{D^{t}\left(x^{t},y^{t}\right)}\right]{\left[\frac{D^{t}\left(x^{t+1},y^{t+1}\right)}{D^{t+1}\left(x^{t+1},y^{t+1}\right)}\frac{D^{t}\left(x^{t},y^{t}\right)}{D^{t+1}\left(x^{t},y^{t}\right)}\right]}^{1/2}$$ Equation 3

in which: D^t^ the distance function that measures efficiency in converting inputs (x^t^) into outputs (y^t^) in period t.

The values of MI, E_MI,_ and T_MI_ refer to measures of change, indicating variation: positive if > 1 (evolution); negative if < 1 (involution); constant if = 1 (stability) .^14^

## RESULTS

The 27 capitals meet the minimum spending (15%) on health^18^ and are large cities, 14 of which are metropolises (> 900,000 inhabitants)^19^. Only Rio de Janeiro, Porto Alegre, São Paulo, and Fortaleza have PHC units under TA (Table 1) and they account for 46% of the population. The others use DA.

There is great heterogeneity, with extreme values hatched, in Table 1. The highest gross domestic product (GDP) *per capita* is in Brasília (R$ 85,661.39), followed by Vitória (R$73,632.55), which has the highest population coverage by PHC (64.20%). Teresina has the highest health expenditure per inhabitant (R$ 1,677.84) and allocates more of its own resources to PHC (42.20%), despite its 22nd place in GDP. Health spending per inhabitant also stands out positively in Campo Grande (R$ 1,535.96), Cuiabá (R$ 1,597.80), and Belo Horizonte (R$ 1,463.70), which contrasts with the nine capitals with values below R$ 710.00. Among the metropolises, the lowest figures were in Salvador (R$ 635.05) and Rio de Janeiro (R$ 630.30). The latter had the highest spending on personnel in the municipal executive branch (56.20%), exceeding the maximum limit (54.00%) stipulated by the LRF. The highest spending per inhabitant on PHC occurred in Florianópolis (R$ 517.22), São Paulo (R$ 403.64), and Recife (R$ 390.40), in contrast to the figures of less than R$ 100.00 in eight capitals. Boa Vista (32.83%) and Macapá (33.66%) were more dependent on revenue (%) from the municipal participation fund (FPM).

We also analyzed the main associations between the indicators in Table 1, in Pearson correlations: strong negative (-0.78) between % FPM and % SS Population and moderate between % FPM and GDP (-0.61); and moderate positive between % SS Population and GDP (0.60). Capitals where the population has a higher income have less dependence on federal financial transfers and greater use of health insurance.

Table 2 shows the capitals ranked by their E_technical_ in 2019, with at least two efficient capitals in each region. Fifteen capitals were efficient (E_technical_ = 1), i.e. they used technology that was better able to convert spending per inhabitant (inputs, X_i_) into PHC results (outputs, Y_i_). Twelve capitals showed weak inefficiency and lower performance in this conversion. The study of the temporal stability of these efficiencies by comparing them with previous years (2016, 2012, 2008) revealed consistency in the maintenance of E_technical_ by the 15 efficient capitals, as well as the 12 weakly inefficient capitals, with annual oscillations in this condition.

**Table 2:** Efficiency scores of capital cities by region of Brazil and Federated Unit (2019, 2016, 2012, and 2008).

Capital	UF	Region	2019				2016	2012	2008
			Level^[^ ^1^ ^]^	E_technical_	E_Global_	Return of scale	E_technical_	E_technical_	E_technical_
Porto Velho	RO	N	Efficient	1	1	Constant	1	1	1
Rio Branco	AC	N	Efficient	1	1	Constant	1	1	1
Macapá	AP	N	Efficient	1	1	Constant	1	1	1
Palmas	TO	N	Efficient	1	0.48	Decreasing	1	1	1
São Luís	MA	NE	Efficient	1	0.61	Decreasing	1	1	1
Teresina	PI	NE	Efficient	1	0.57	Decreasing	1	1	1
Maceio	AL	NE	Efficient	1	1	Constant	1	1	1
Salvador	BA	NE	Efficient	1	1	Constant	1	1	0.99
Belo Horizonte	MG	SE	Efficient	1	0.36	Decreasing	1	1	1
Victoria	ES	SE	Efficient	1	1	Constant	1	1	1
Curitiba	PR	S	Efficient	1	0.59	Decreasing	1	1	1
Florianópolis	SC	S	Efficient	1	1	Constant	1	1	1
Cuiabá	MT	CW	Efficient	1	0.48	Decreasing	1	1	0.8
Goiânia	GO	CW	Efficient	1	0.61	Decreasing	1	1	0.94
Brasilia	DF	CW	Efficient	1	1	Constant	1	1	1
João Pessoa	PB	NE	Weak Inefficiency	0.98	0.65	Decreasing	1	0.87	1
Manaus	AM	N	Weak Inefficiency	0.97	0.94	Decreasing	1	1	1
Bethlehem	PA	N	Weak Inefficiency	0.94	0.66	Decreasing	0.85	1	0.88
Boa Vista	RR	N	Weak Inefficiency	0.94	0.74	Decreasing	1	1	1
Recife	PE	NE	Weak Inefficiency	0.91	0.58	Decreasing	0.84	1	1
São Paulo	SP	SE	Weak Inefficiency	0.91	0.48	Decreasing	0.9	0.92	0.99
Aracaju	SE	NE	Weak Inefficiency	0.91	0.69	Decreasing	0.92	1	1
Porto Alegre	RS	S	Weak Inefficiency	0.89	0.76	Decreasing	0.84	0.97	0.94
Christmas	RN	NE	Weak Inefficiency	0.87	0.73	Decreasing	0.9	0.95	0.84
Rio de Janeiro	RJ	SE	Weak Inefficiency	0.85	0.68	Decreasing	0.83	0.79	0.87
Fortaleza	EC	NE	Weak Inefficiency	0.84	0.62	Decreasing	0.89	0.94	0.86
Campo Grande	MS	CW	Weak Inefficiency	0.81	0.39	Decreasing	0.90	1	0.95
			Average (n = 27)	0.96			0.96	0.98	0.97
			Average DA (n = 23)	0.99			0.98	0.99	0.98
			Average OS/FEDP (n = 4)	0.87			0.87	0.9	0.91

UF: Federated Unit; E_technical_: efficiency obtained by VRS-O DEA; E_global_: efficiency obtained by DEA CRS-O; DA: direct administration; OS/FEDP: social organizations or state public foundation under private law.

Note: when interpreting the scores we consider: efficient (1); weak inefficiency (0.8 to 1); moderate inefficiency (0.6 to 0.8); strong inefficiency (less than 0.6).

The returns to scale of the PHC processes in each capital in 2019 (Table 2) were calculated by estimating E_Global_. Among the efficient capitals, eight operate on the ideal transformation scale (constant) when transforming inputs into outputs. The other seven do not operate on the most productive scale (decreasing) and could obtain even more *outputs* (Y_i_). As expected, all the inefficient capitals operate on a non-ideal (decreasing) scale when producing outputs, i.e. if there is a 10% increase in *per capita* spending (X_i_), a smaller percentage improvement in outputs is expected.

The E_technical_ scores in Table 2 were studied separately for the capitals with PHC units under TA, which in 2019 had an average E_technical_ score of 0.87, lower than that of the capitals that adopt a TA score of 0.99. This difference has increased over the 12 years.

Table 2 also shows the cross-sectional analyses of E_technical_ for the years 2008, 2012 and 2016. Twelve capitals in HC remained efficient in all years, while the municipalities in HC consistently showed weak inefficiency in PHC, i.e. in the period studied, the relationship between investments in PHC and the results obtained was lower than in the efficient capitals.

The DEA also provides the values of the indicators in the estimation of targets for the outputs (Y_i_) of the non-efficient capitals (Table 3). For example, in order to be efficient, São Paulo would need to simultaneously: increase PHC coverage by 6.1% and the proportion of tuberculosis cured by 7.2%; and reduce the ICSAB (1.2), infant mortality rate (1.0), premature mortality rate (164.9), maternal mortality rate (13.5) and congenital syphilis incidence rate (3.8). These targets could help the search for greater efficiency.

**Table 3:** Indicator values and targets, by non-efficient capital, in 2019.

Capital	C_AB		ICSAB		Tx_M_Inf		P_Cure_Tb		T_M_Prem		T_M_Mat		TX_Syp	
	A	M	A	M	A	M	A	M	A	M	A	M	A	M
João Pessoa	92.9	94.7	14.8	14.1	12.7	9.4	66.8	68.1	721.8	548.7	57.9	45.5	9.4	5.1
Manaus	46.3	69.1	24.0	14.0	13.8	13.4	69.9	77.8	524.2	509.5	68.5	47.5	16.0	5.1
Bethlehem	39.4	51.2	21.9	10.2	15.5	13.6	70.2	74.6	649.1	558.6	69.9	46.2	6.4	5.1
Boa Vista	61.1	66.8	28.0	14.4	14.2	12.1	71.4	76.3	530.3	496.2	56.0	52.4	4.6	4.3
Recife	64.8	70.9	14.3	12.9	11.5	10.5	64.9	71.0	811.3	543.3	28.4	26.0	26.9	4.5
São Paulo	62.6	68.7	13.3	12.1	11.2	10.2	73.8	81	739.8	574.9	62.4	48.9	7.5	3.7
Aracaju	70.7	78.0	14.4	13.1	16.9	14.2	66.8	73.7	579.3	525.6	46.4	42.1	16.4	5.9
Porto Alegre	67.9	75.9	17.1	13.2	8.7	7.8	52.6	58.7	928.5	644.5	24.2	21.7	25.4	6.5
Christmas	52.5	60.6	13.0	11.3	12.9	10.5	57.8	68.6	706.2	612.5	37.6	32.6	26.5	6.2
Rio de Janeiro	54.9	64.9	13.6	11.5	12.2	10.3	65.0	77.0	926.4	585.4	80.7	47.1	14.8	4.3
Fortaleza	44.2	60.7	16.9	14.2	11.7	9.8	60.3	71.6	617.9	520.2	40.1	33.7	16.0	4.7
Campo Grande	48.4	64.7	14.8	12.1	9.3	7.5	51.1	62.8	706.5	575.5	49.9	22.5	8.5	5.4

A: current values obtained; M: target to be achieved; C_PC: population coverage estimated by primary care teams; ICSAB: proportion of hospitalizations for primary care sensitive conditions; Tx_M_Inf: infant mortality rate; P_Cure_Tb: proportion of cure in new cases of pulmonary tuberculosis; Tx_M_Prem: premature mortality rate (< 70 years) for the four main chronic non-communicable diseases as a whole; T_M_Mat: maternal mortality rate; T_Syp: incidence rate of congenital syphilis cases.

The results of the changes in productivity by comparing the MI and its components (E_MI_, T_MI_), in a dynamic perspective of the change between successive efficiency frontiers (2008 and 2019) were grouped according to the management models (DA and TA) (Table 4).

**Table 4:** Changes in the Malmquist index and its components between 2008 and 2019, by capital groupings according to management model.

Statistics	General			Direct administration			Third-party administration		
	MI	E_MI_	T_MI_	MI	E_MI_	T_MI_	MI	E_MI_	T_MI_
Geometric mean	0.70	1.02	0.69	0.66	1.04	0.64	0.94	0.90	1.04
Average	0.84	1.05	0.80	0.82	1.07	0.76	0.97	0.93	1.05
Coefficient of variation	45.65	25.34	39.69	49.00	25.02	42.93	23.04	23.23	4.05
Minimum	0.09	0.58	0.09	0.09	0.58	0.09	0.65	0.66	0.99
Maximum	1.59	1.61	1.15	1.59	1.61	1.15	1.27	1.25	1.10

Note: for the calculation of the values, the Yi of 2008 were corrected by the Extended National Consumer Price Index for 2019.

MI: Malmquist Index (MI) and its components: Efficiency (EMI) and Technology (TMI).

There was a reduction in sectoral productivity (General), with $\mathrm{MI}=0.70\left(0.69^{*}1.02\right)$ , which resulted from the technological involution (< 1) (T_MI_ = 0.69) and the very small positive change (> 1) in efficiency (E_MI_ = 1.02). As a result, on average, the PHC evolved (MI) in the conversion of inputs (X_i_) into outputs (Y_i_). The evolution of E_MI_ expresses the use of the ideal scale (adaptation to the most productive size) and the gain in technical efficiency when converting inputs into outputs (X_i_ used in 2008 generated less Y_i_ in 2019).

There was also an average decline in MI in both forms of PHC management. PHC under DA evolved in Efficiency (E_MI_) and evolved in technology (T_MI_) while under TA it evolved in Efficiency (E_MI_) and evolved in technology (T_MI_). The negative change in T_IM_ scores is not the result of empirical observation of technology in the capitals, but of comparing their distance from the VRS-O DEA efficiency frontier, measured at times t (2008) and t+1 (2019). The smaller variation (CV, max, min) in the scores under TA indicates cities that are more homogeneous in their provision of PHC.

## DISCUSSION

The analysis of PHC efficiency in Brazilian capitals in the 2008–2019 period resulted in 15 capitals with efficient DA, while another 12 showed weak inefficiency. Among the inefficient ones are capitals with administration transferred to third parties (AT).

There is a predominance of TA in Rio de Janeiro, Porto Alegre, São Paulo, and Fortaleza, which includes the two most populous capitals. This result is in line with a previous survey^19^, which indicated that municipal health establishments managed by third parties predominate in the Southeast (72.4%), followed by the South (15.2%) and Northeast (8.0%). The Foundations in the South and the OS in the Southeast stand out, accounting for 83.3% of outsourced institutions.

Health management by OS has a significant presence in cities or states with greater economic power, with relevance to their origin and the largest number in the state of São Paulo^20^, confirming their entrepreneurial nature and location in the country’s main economic hubs. Another study^21^ corroborates this finding by demonstrating the geographical expansion of the country’s ten largest OSs, present in 17 states, with a concentration in the south-southeast axis, with the greatest economic dynamism. Similarly, the capitals with the highest percentage of health insurance coverage are in the seven capitals of these regions.

All the capitals invest or exceed the minimum percentage of resources invested in health, which is consistent with other authors. Since 2004, all Brazilian municipalities have exceeded the minimum percentage (15%), with an average of 22.5% in 2017.^22^

The legal limit on personnel spending in the municipal executive branch (54%) set by the LRF is one of the main arguments for implementing TA^23^, especially in the PHC. In Rio de Janeiro, outsourcing did not reduce the percentage of 56.2%. There is also a reduction^23^ in fiscal effort in Rio, which invested 25.5% of its own revenue in 2016, with a subsequent reduction starting in 2017, when it invested only R$ 3.31 *per capita/*day in health, below the national average (R$ 3.38 *per capita/*day) .^24^

The highest *per capita* spending on PHC in 2020 was made in Florianópolis (R$ 517.22) and São Paulo (R$ 403.64), which may suggest a low influence of the management model on the prioritization of PHC in health spending. However, population coverage of PHC in Florianópolis has historically been higher than in São Paulo.^5^

Over time, there was stability in the efficiency scores for 12 of the 15 capitals under DA. This regularity is in line with a study which indicated Florianópolis, Belo Horizonte, and Curitiba as national references for a strong PHC network with an emphasis on the ESF^25^. Similarly, the fact that São Paulo was not efficient in all the years studied reaffirms an analysis carried out in 2015^26^, that the experience of management by the private sector in São Paulo does not achieve greater efficiency by combating patrimonialism.

The E_technical_ scores for the capitals (Table 2) were obtained under different health investment conditions. Considering values adjusted for 2019, PHC under DA reduced average spending from R$ 232.86/hab to R$ 153.08/hab between 2008 and 2019 (-52.1%), while capitals with TA increased from R$ 127.00/hab to R$ 211.94/hab (+66.9%). There is a disproportion in investments related to the decreasing production scales and inefficiencies of the capitals under TA, which have higher *per capita* spending (X_i_) without a corresponding improvement in health status (Y_i_). It should be reiterated that the search for efficiency in public PHC is limited by the continued stifling of funding.^9,17^

Analysis of the change in productivity (MI and its components E_MI_, T_MI_) between 2008 and 2019 also showed differences between PHC management models. Both showed a decline in average productivity (MI). In the capitals under DA, this effect was greater due to the technological decline (T_MI_) and the simultaneous increase in efficiency when generating results (E_MI_ = 1.04), due to the improvement in pure technical and scale efficiency. Conversely, in the capitals under TA (Fortaleza, Rio de Janeiro, São Paulo, and Porto Alegre) there was technological evolution (T_MI_ = 1.04), related to the capitals’ ability to adapt to available technology, but with a reduction in efficiency. These results are consistent with the reduction in investments and may also be due to the greater heterogeneity between the capitals under TA.

It is worth considering that productive efficiency may not be effective in meeting health demands^9^. This issue is important in the context of evaluations of agreement processes between federated entities, where counting the number of indicators with targets met by municipalities may allow them to be compared, but not to assume their efficiency.

Our results are in line with studies carried out in other contexts. Better performance in philanthropic hospitals compared to private for-profit hospitals^3^; no proof of the superiority of alternative models to DA, and there is still little evidence of the positive impacts of management in public-private arrangements^27,28^. Furthermore, the privatization of the Unified Health System (SUS) as a solution for greater efficiency has been treated as a myth.^9^

The international literature review also found no evidence of greater efficiency in public or private provision in the health sector. Increased privatization of the NHS by subcontracting services to for-profit companies has led to a decline in the quality of health care, with an increase in avoidable mortality^29^, just as there has been a worsening of health care and outcomes in other high-income countries.^30^

The limitations of this study may stem from the method and data used. The choice of model adopted (VRS-O DEA and Malmquist index) produced consistent results, but we would point out that its efficiency frontiers (vertical and horizontal) can generate efficiencies that do not perfectly meet the Pareto-Koopmans concept. It would have been possible to use more flexible and dynamic DEA models, but they are not without intrinsic limitations, especially the assumptions of additivity, divisibility, proportionality and certainty. As for the variables, the number of DMUs did not allow the use of stochastic forms of DEA. As for the data, the indicators used may influence the estimated efficiency due to their incompleteness in representing PHC and, furthermore, although there is a consistent logical relationship between the inputs and products used, efficiency may be influenced by competing socio-economic factors exogenous to PHC, which were not studied.

## CONCLUSIONS

Achieving efficiency does not necessarily mean meeting health needs. The results indicate that PHC units under DA are more efficient than those under TA and do not show evidence of efficiency gains in the PHC of Brazilian capitals due to the adoption of alternative management models. This contradicts the benefits disseminated by neoliberal thinking and incorporated by the new public management and also contrasts with the assumption that there is greater efficiency in the provision of public services with the adoption of market fundamentals and the inclusion of private institutions to qualify social policies.
