# Supplementary material for: Efficiency of primary care in Brazilian capitals and management models: 2008–2019
Source: Rev Saude Publica. 2025 May 2;59:e6. doi: 10.11606/s1518-8787.2025059006346 (PMC12002741; doi:10.11606/s1518-8787.2025059006346)
Supplement: Translation

# Translation: Eficiência da atenção primária nas capitais brasileiras e modelos de gestão: 2008–2019

**Authors:** André Luis Paes Ramos, Marismary Horsth De Seta, Marcelo Battesini

**Affiliations:** I Fundação Oswaldo Cruz. Escola Nacional de Saúde Pública Sergio Arouca. Programa de Pós-graduação em Saúde Pública. Rio de Janeiro, RJ, Brasil; II Universidade Federal de Santa Maria. Programa de Pós-Graduação em Gestão de Organizações Públicas. Santa Maria, RS, Brasil

**Keywords:** Atenção Primária à Saúde, Serviços Terceirizados, Avaliação em Saúde, Eficiência

## Abstract

**OBJETIVO:**

Analisar a eficiência na atenção primária à saúde das capitais brasileiras no período 2008–2019, considerando o modelo de gestão: administração pública direta ou administração cedida a terceiros.

**MÉTODOS:**

Estudo de propósito avaliativo, objetivo analítico, com dados secundários de acesso público, analisados em recorte transversal (2019) e longitudinal (2008, 2012, 2016 e 2019). Para caracterizar as cidades e sua atenção primária à saúde, utilizaram-se dados demográficos, socioeconômicos e sete indicadores relacionados à atenção primária à saúde (cobertura em atenção básica, internações por condições sensíveis à atenção básica, cura de tuberculose, mortalidade infantil, materna e prematura por condições crônicas, incidência de sífilis congênita). A esses indicadores foram acrescidos os de gastos em saúde e em atenção primária à saúde e realizou-se a análise envoltória de dados voltada a outputs (sem admitir redução de gastos) para cálculo da eficiência em 2008, 2012, 2016 e 2019. Com o índice de Malmquist, buscou-se identificar possíveis ganhos de produtividade entre 2008 e 2019.

**RESULTADOS:**

Quatro capitais identificadas como de administração cedida a terceiros (São Paulo, Rio de Janeiro, Porto Alegre e Fortaleza) não obtiveram maior eficiência perante a administração pública direta, tampouco evoluíram quando comparados os seus próprios resultados no tempo. Em 2019, e na abordagem longitudinal, apenas capitais de gestão por administração pública direta obtiveram índice máximo de eficiência relativa na comparação entre as capitais. Doze capitais em administração pública direta mantiveram-se eficientes em todos os anos, ao passo que as em administração cedida a terceiros apresentaram, consistentemente, ineficiência fraca em atenção primária à saúde, ou seja, no período estudado, a relação entre investimentos na atenção primária à saúde e resultados obtidos é inferior ao alcançado pelas capitais eficientes.

**CONCLUSÕES:**

Não encontramos evidências de ganhos de eficiência com a adoção de administração cedida a terceiros. Ressalte-se que a eficiência produtiva não representa, necessariamente, o atendimento de necessidades de saúde.

## INTRODUÇÃO

Países com sistemas de saúde universais investem fortemente na Atenção Primária à Saúde (APS) para acesso ao sistema e resolução de necessidades mais comuns^1,2^.

No Brasil, cabem principalmente aos municípios a gestão e a execução das políticas de APS. As capitais dos estados destacam-se pelas condições estruturais, econômicas, políticas e sociais, e por serem polos regionais de referência, não apenas para os municípios circundantes.

Há diversos projetos em disputa sobre a melhor forma para gerir e prestar serviços em saúde, estando pautado o debate sobre a maior ou menor presença do Estado na provisão de políticas sociais. Um argumento seria a suposta maior eficiência do setor privado sobre o setor estatal^3^. Gestores públicos brasileiros apontam as leis de licitações e a Lei de Responsabilidade Fiscal (LRF) como constrangimentos para a gestão pública exclusiva, tornando necessário o uso de modelos alternativos de gestão, mais flexíveis para compras e contratação de pessoal^4^.

Alternativas à administração pública exclusiva proliferam: fundações estatais de direito público ou privado, empresas públicas, organizações não governamentais, organizações sociais (OS), Organizações da Sociedade Civil de Direito Público, Organizações da Sociedade Civil, Serviço Social Autônomo. São frequentes os arranjos mistos, em que parte das unidades de saúde é gerida pela administração pública direta ou indireta, e outra parte é terceirizada para instituições privadas; na mesma unidade de saúde podem coexistir distintos modelos.

Criadas em 1998, as OS representam a principal estratégia de terceirização da administração de unidades públicas de saúde, mas sua expansão não foi acompanhada de estudos sobre se a propalada eficiência fora ou não alcançada, principalmente na APS. Estudos sobre o desempenho da atenção hospitalar gerida por OS ou administração pública comparam hospitais por indicadores de cada estabelecimento, sem focalizar resultados e impactos sobre a saúde da população assistida no sistema em que estão inseridos.

Trabalhos recentes^5,6^ analisaram o desempenho da APS – compreendido como a medida em que o sistema de saúde das capitais brasileiras atinge os objetivos propostos^7^. Contudo, a eficiência não fora analisada e os gastos realizados para o alcance dos resultados não foram considerados.

A eficiência, um princípio constitucional para a administração pública^8^, consta nos Descritores em Ciências da Saúde (DeCs) como a razão entre esforço e recursos empreendidos. A relação eficiente entre resultados obtidos e a quantidade de insumos utilizados (função produção) não é facilmente determinada em saúde pública, e demanda a análise das melhores práticas de referências virtuosas (*benchmarks*), que delimitam uma fronteira limítrofe para unidades que compartilham condições similares^9^. No entanto, o uso de medidas de eficiência não deve servir para diminuição na alocação de recursos públicos, dada a sua contribuição minoritária para a composição do gasto em saúde no Brasil, predominantemente privado^9^.

Este estudo objetiva analisar a eficiência da APS nas capitais brasileiras no período 2008–2019, a partir de duas questões. A primeira, se as capitais são eficientes ao ofertar a APS; e a segunda, se o modelo de gestão da APS adotado impactou a eficiência.

## MÉTODOS

Estudo de propósito avaliativo, objetivo analítico, com dados secundários, de acesso público, analisados em recorte transversal (2019) e longitudinal (2008, 2012, 2016 e 2019).

Pesquisa realizada em duas fases. Na primeira, a APS nas 27 capitais foi caracterizada mediante indicadores usuais^5,10,11^ (Tabela 1) referentes a dados populacionais, modelo de gestão, gasto e arrecadação públicos e percentual de população coberta por planos de saúde suplementar (população em SS), priorizando a informação mais recente (entre 2020 e 2021). Estimaram-se os valores *per capita* para reduzir a influência de disparidades socioeconômicas e de porte populacional. Dois destes indicadores foram utilizados também na segunda fase (gasto em saúde e gasto em APS).

**Tabela 1:** Caracterização das capitais segundo indicadores selecionados (2018, 2020 e 2021).

Capital/UF	População^b^	Unidades APS sob AT^a^	PIB (R$/hab)^b^	Gasto em Saúde (R$/hab)^c^	Gasto em APS (R$)^c^	% FPM^c^	% Recursos próprios em Saúde^c^	% População em SS^d^	% Gastos Pessoais LRF^c^
	2020	2021	2018	2020	2020	2020	2020	(Dez 2020)	2020
Porto Velho (RO)	539.354	0	32.042,66	706,62	52,12	17,68	23,1	18,2	50,79
Rio Branco (AC)	413.418	0	22.287,70	319,46	267,98	35,50	16,1	11,2	41,92
Manaus (AM)	2.219.580	0	36.445,75	503,11	163,07	10,83	23,6	27,6	40,86
Boa Vista (RR)	419.652	0	26.752,67	593,62	154,42	32,83	19,3	9,8	43,14
Belém (PA)	1.499.641	0	21.191,47	514,26	78,92	15,52	27,1	28,6	45,25
Macapá (AP)	512.902	0	22.181,72	438,6	206,17	33,66	17,5	12,8	50,35
Palmas (TO)	306.296	0	32.293,89	841,52	264,63	20,50	21,1	21,9	48,46
São Luís (MA)	1.108.975	0	30.699,57	819,87	58,49	17,42	21,6	29,0	44,78
Teresina (PI)	868.075	0	24.333,00	1.677,84	127,39	19,56	42,2	30,8	47,17
Fortaleza (CE)	2.686.612	20	25.356,73	958,16	172,42	12,84	28,7	36,4	46,03
Natal RN)	890.480	0	27.122,37	836,78	41,25	13,55	30,7	37,1	50,62
João Pessoa (PB)	817.511	0	25.035,80	880,55	150,46	14,12	21,1	31,9	47,58
Recife (PE)	1.653.461	0	31.994,38	936,57	390,40	10,58	23,2	39,9	46,99
Maceió (AL)	1.025.360	0	22.126,34	765,92	59,01	16,62	21,3	29,2	43,72
Aracaju (SE)	664.908	0	26.622,38	757,37	202,57	17,06	22,3	39,5	46,46
Salvador (BA)	2.886.698	1	22.232,68	635,05	30,04	12,66	22,2	29,7	36,93
Belo Horizonte (MG)	2.521.564	0	36.759,66	1.463,70	317,00	4,86	22,9	48,7	41,85
Vitória (ES)	365.855	0	73.632,55	756,80	308,54	11,73	18,3	64,2	46,31
Rio de Janeiro (RJ)	6.747.815	217	54.426,08	630,30	132,45	1,56	16,8	47,5	56,24
São Paulo (SP)	12.325.232	74	58.691,90	1.042,83	403,64	0,54	21,8	50,5	33,58
Curitiba (PR)	1.948.626	0	45.458,29	1.030,94	350,04	4,57	20,1	55,3	42,25
Florianópolis (SC)	508.826	0	42.719,16	705,89	517,22	7,61	17,6	42,0	48,68
Porto Alegre (RS)	1.488.252	109	52.149,66	1.180,80	196,45	4,27	18,1	43,6	41,81
Campo Grande (MS)	906.092	0	32.942,46	1.535,96	263,26	4,35	25,4	30,5	50,39
Cuiabá (MT)	617.848	0	39.043,32	1.597,80	101,29	5,88	34,7	40,7	45,63
Goiânia (GO)	1.536.097	0	33.004,01	1.037,47	55,66	7,04	19,9	38,9	44,12
Brasília (DF)	3.055.149	0	85.661,39	1.137,83	47,70	0,74	17,75	34,3	42,05

UF: Unidade Federada – população utilizada pelo Tribunal de Contas da União; APS: atenção primária à saúde – estabelecimentos sob administração de terceiros; PIB: Produto interno bruto; FPM: Fundo de participação dos municípios no total das Receitas; SS: saúde suplementar – cobertura por planos; LRF: Lei de Responsabilidade Fiscal.

Fontes: ^a^Cadastro Nacional de Estabelecimentos de Saúde (CNES); ^b^Instituto Brasileiro de Geografia e Estatística (IBGE); ^c^Sistema de Informações sobre Orçamentos Públicos em Saúde - despesas liquidadas; ^d^Agência Nacional de Saúde Suplementar (ANS).

Considerando que a qualidade da administração dos serviços de saúde pode impactar positivamente seu desempenho^9^, os modelos predominantes de gestão da APS de cada capital foram identificados a partir de informações obtidas com gestores locais^5^, com busca bibliográfica para atualização. Esse procedimento gerou as categorias: administração pública direta exclusiva (AD) e administração concedida a terceiros (AT). Os indicadores foram analisados mediante estatística descritiva e correlação de Pearson.

Na segunda fase, estudaram-se as eficiências na gestão da APS das capitais, por meio de análise envoltória de dados (AED ou DEA, *data envelopment analysis*), método multidimensional não paramétrico utilizado para estimar a eficiência de unidades tomadoras de decisão (DMU, *decision making units*), com base em programação linear matemática^12^. Além de gasto por habitante na função saúde e em APS, foram utilizados como variáveis indicadores de saúde presentes no Pacto de Diretrizes e Metas (PDM) 2013–2015^13^ (Quadro), calculados a partir de dados de bases nacionais. As análises e a solução dos modelos AED foram realizadas no ambiente R-Studio (R Core Team, 2022), com o auxílio do pacote *benchmarking* e suas funções.

**Quadro:** Inputs (Xi) e Outputs (Yi) utilizados na aplicação de análise envoltória de dados (AED).

Indicador	Descrição	Fonte	Variável
Gasto por habitante na Função Saúde	Montante de recursos destinados à macro função Saúde	SIOPS/IBGE	X_1_
Gasto por habitante na Atenção Básica	Montante de recursos destinados à subfunção Atenção Básica	SIOPS/IBGE	X_2_
Cobertura das equipes de AB	Recursos humanos da APS, expressando a potencial de oferta de ações e serviços básicos e o acesso a ela	E-gestor AB	Y_1_
Proporção de ICSAB	ICSAB, expressando o risco de hospitalização desnecessária que pode ser diminuído por ações efetivas da APS	SIH	Y_2_
Taxa de mortalidade infantil	É um dos principais indicadores de saúde pública. Revela as condições de saúde materno-infantil e expressa a situação de saúde de uma comunidade e as desigualdades entre grupos sociais e regiões	SIM / SINASC	Y_3_
Proporção de cura de novos casos de tuberculose pulmonar	Indica êxito no tratamento de tuberculose, dificuldades no monitoramento dos casos identificados e redução na transmissão	Sinan	Y_4_
Taxa de mortalidade prematura por-DCNT	Monitora o impacto das políticas públicas na prevenção e controle das principais DCNT (aparelho circulatório, câncer, diabetes e respiratórias crônicas) e seus fatores de risco em pessoas com menos de 70 anos	SIM/IBGE	Y_5_
Taxa de mortalidade materna	Expressa as mortes de mulheres em idade fértil por causas ligadas à gravidez, ao parto e ao puerpério, geralmente passíveis de prevenção e evitáveis	SIM / SINASC	Y_6_
Taxa de incidência de sífilis congênita	Indica a qualidade do pré-natal, dado a sífilis poder ser diagnosticada e tratada durante a gestação	Sinan	Y_7_

APS: atenção primária à saúde; AB: atenção básica; ICSAB: internações por condições sensíveis à atenção básica; SIOPS: Sistema de Informações sobre Orçamentos Públicos em Saúde; IBGE: Instituto Brasileiro de Geografia e Estatística; E-gestor AB: Informação e Gestão da Atenção Básica; SIH: Sistema de Informações Hospitalares; SIM: Sistema de Informação sobre Mortalidade; SINASC: Sistema de Informações sobre Nascidos Vivos; Sinan: Sistema de Informação de Agravos de Notificação; DCNT: doenças crônicas não transmissíveis.

A AED é baseada na produtividade das DMU analisadas, estimada pela razão entre resultados obtidos e os recursos utilizados. A eficiência de uma DMU é obtida pela solução do problema de programação linear que otimiza razão $\theta =\text{ output virtual/input virtual }=\left[v_{1}y_{1}+v_{2}y_{2}+\ldots +v_{n}y_{n}\right]/\left[u_{1}x_{1}+u_{2}x_{2}+\ldots +u_{m}x_{m}\right]$ , com θ ≥ 1, sendo v_i_ e u_i_ os pesos ótimos (v_i_ e u_i_) obtidos, com n variáveis de *output* e m de *input* ^14^, como resultado da comparação entre a produtividade de uma DMU em relação à produtividade máxima definida por uma fronteira latente.

Em geral, a AED minimiza *inputs* e maximiza *outputs* buscando mais eficiência ou melhor performance^15^, e as DMU eficientes formam uma fronteira que serve de referência às ineficientes. A eficiência resulta da comparação entre as distintas habilidades em converter *inputs* em *outputs* (produtividade) e a fronteira de eficiência endógena é identificada por um modelo de programação matemática linear^16^. Os passos utilizados são: definir DMU; selecionar variáveis; definir modelo; obter e apresentar resultados^12^. Adicionalmente, estimamos a mudança de produtividade ao longo do tempo.

As 27 capitais foram tomadas como DMU, por apresentarem condições similares de oferta e serem referências locais em APS. As variáveis de *input* (X_i_, recursos), gastos públicos por habitante na função saúde (X_1_) e na Atenção Básica (X_2_), entendida neste estudo como sinônimo de APS, expressam investimento em saúde e o grau de prioridade atribuído pela gestão ao primeiro nível de atenção. As variáveis utilizadas como *output* (Yi) incluíram indicadores de produção (produtos intermediários) e de efetividade e desfechos (*outcomes*)^9^, constam no Quadro.

As variáveis de *output* Y_2_, Y_3_, Y_5_, Y_6_ e Y_7_ (menor-é-melhor) foram tomadas em seu inverso nas análises AED para assumirem a mesma direção das demais (maior-é-melhor) na otimização.

Foi utilizado o modelo AED de retorno variável orientado ao *output* (VRS−O), relacionado à forma como a fronteira de eficiência é definida e a orientação se refere à direção escolhida para estimar a eficiência das DMU ineficientes^16^. Dois modelos matemáticos são utilizados na AED VRS−O, representados nas Equações 1 e 2^12^. Na primeira, é apresentado o primal na forma do envelope (definição da fronteira) e na segunda o dual na forma dos multiplicadores expressos algebricamente (definição dos pesos).


$$\max\theta_{0}|\text{ sujeito à: }X\lambda \leq x_{0};\theta y_{0}-Y\lambda \leq 0;e\lambda =1;\lambda \geq 0$$ Equação 1

nas quais, q_0_ é a eficiência da DMU sob análise; X= {x_i_} é um conjunto de variáveis de input; Y= {y_j_} é um conjunto de variáveis de output; x_0_ e y_0_ são as variáveis de *input* e de *output* da DMU sob análise; l é o vetor de pesos da DMU sob análise; e é um vetor com elementos iguais a 1; e l = 1 impõe a condição de convexidade.


$$\min\left[\left({\mathrm{vx}}_{0}-v_{0}\right)/{\mathrm{uy}}_{0}\right]|\text{ sujeito à: }\left[\left({\mathrm{vx}}_{i}-v_{0}\right)/{\mathrm{uy}}_{j}\right]\geq 1$$ Equação 2

nas quais, x_i_ e y_j_ são variáveis de *input* e *output*; e v_i_ e u_i_ os pesos ótimos.

O modelo VRS−O permite estimar as eficiências técnicas (E_técnica_) das DMU, a partir de uma envoltória convexa definida por trechos, buscando maximizar os níveis de *outputs* considerando o atual consumo de *inputs* ^12^. A orientação a *outputs* é compatível com pesquisas na área de saúde pública, cujo orçamento é limitado^9,17^ e não se almeja a sua redução, apesar de ser esperada progressiva melhoria dos indicadores de APS.

Os escores de E_técnica_ estimados pelo modelo AED VRS expressam um componente da eficiência global (E_global_), que também inclui a eficiência de escala E_escala_ = E_técnica_ / E_global_^12^. Assim, para identificar se uma DMU opera em sua escala mais produtiva (economia de escala), é necessário estimar o modelo AED CRS^14^.

O modelo AED de retorno variável orientado ao *output* (AED VRS−O) foi estimado para os anos de 2019, 2016, 2012 e 2008 (análises transversais). Complementarmente, foi estimada a E_global_ pelo modelo AED de retorno constante (AED CRS−O) para identificar a E_escala_ das DMU no ano de 2019.

As análises transversais geram resultados pontuais sobre a eficiência relativa entre as DMU, sem considerar que o fenômeno investigado possa evoluir ou involuir no tempo. Para observar essas modificações, foi estimado o índice de Malmquist (IM), entre 2008 e 2019 (análise longitudinal). O IM é uma medida global de mudança das DMU no tempo (progresso ou retrocesso) e expressa os ganhos de produtividade entre os tempos t e t+1^15^. IM é definido como o produto de seus componentes (IM = E_IM_ *T_IM_), que expressam a mudança na Eficiência (E_IM_, *catch-up effect*) e a mudança na Tecnologia (T_IM_, *frontier-shift effect*), sendo calculado pela Equação 3^16^.


$$\mathrm{per}IM^{t+1}\left(x^{t+1},y^{t+1},x^{t},y^{t}\right)=\left[\frac{D^{t+1}\left(x^{t+1},y^{t+1}\right)}{D^{t}\left(x^{t},y^{t}\right)}\right]{\left[\frac{D^{t}\left(x^{t+1},y^{t+1}\right)}{D^{t+1}\left(x^{t+1},y^{t+1}\right)}\frac{D^{t}\left(x^{t},y^{t}\right)}{D^{t+1}\left(x^{t},y^{t}\right)}\right]}^{1/2}$$ Equação 3

na qual: D^t^ a função distância que mensura a eficiência na conversão de *inputs* (x^t^) em *outputs* (y^t^) no período t.

Os valores do IM, E_IM_ e T_IM_ referem-se a medidas de mudança, indicando variação: positiva se > 1 (evolução); negativa se < 1 (involução); constante se = 1 (estabilidade)^14^.

## RESULTADOS

As 27 capitais executam os gastos mínimos (15%) em saúde^18^ e são cidades de grande porte, sendo 14 metrópoles (> 900 mil habitantes)^19^. Apenas Rio de Janeiro, Porto Alegre, São Paulo e Fortaleza apresentam unidades da APS sob AT (Tabela 1) e totalizam 46% da população de todas as capitais. As demais adotam AD.

Há grande heterogeneidade, com valores extremos apresentados na Tabela 1. O maior produto interno bruto (PIB) *per capita* ocorre em Brasília (R$ 85.661,39), seguida de Vitória (R$ 73.632,55), que possui a maior cobertura populacional pela SS (64,20%). Teresina apresenta o maior gasto em saúde por habitante (R$ 1.677,84) e aloca mais recursos próprios à APS (42,20%), apesar da 22ª posição no PIB. Também se destacam positivamente os gastos em saúde por habitante em Campo Grande (R$ 1.535,96), Cuiabá (R$ 1.597,80) e Belo Horizonte (R$ 1.463,70), que contrastam com as nove capitais com valores abaixo de R$ 710,00. Dentre as metrópoles, os menores valores ocorrem em Salvador (R$ 635,05) e no Rio de Janeiro (R$ 630,30). Este apresentou o maior gasto com pessoal no poder executivo municipal (56,20%), ultrapassando o limite máximo (54,00%) estipulado pela LRF. Os maiores gastos por habitante em APS ocorreram em Florianópolis (R$ 517,22), São Paulo (R$ 403,64) e Recife (R$ 390,40), discordante dos valores inferiores a R$ 100,00 em oito capitais. Boa Vista (32,83%) e Macapá (33,66%) apresentaram maior dependência de receitas (%) do fundo de participação dos municípios (FPM).

Analisamos também as principais associações entre os indicadores da Tabela 1, em correlações de Pearson: negativa forte (-0,78) entre o % FPM e % População em SS e moderada entre % FPM e PIB (-0,61); e positiva moderada entre % População em SS e PIB (0,60). Capitais onde a população tem maior renda apresentam menor dependência de repasses financeiros federais e maior utilização de planos de saúde.

A Tabela 2 apresenta as 27 capitais ordenadas por suas E_técnica_ em 2019, existindo pelo menos duas capitais eficientes em cada uma das cinco regiões do país. Quinze capitais foram eficientes (E_técnica_ = 1), ou seja, utilizaram tecnologia mais capaz de converter gasto por habitante (*inputs*, X_i_) em resultados da APS (*outputs*, Y_i_). Doze capitais apresentaram ineficiência fraca e menor desempenho nessa conversão. O estudo da estabilidade temporal dessas eficiências ao compará-las com anos anteriores (2016, 2012, 2008) revelou consistência na manutenção das E_técnica_ pelas 15 capitais eficientes, assim como, das 12 capitais fracamente ineficientes, com oscilações anuais dessa condição.

**Tabela 2:** Escores de eficiência das capitais por região do Brasil e Unidade Federada (2019, 2016, 2012 e 2008).

Capital	UF	Região	2019				2016	2012	2008
			Nível^[^ ^1^ ^]^	E_Técnica_	E_Global_	Retorno de escala	E_Técnica_	E_Técnica_	E_Técnica_
Porto Velho	RO	N	Eficiente	1	1	Constante	1	1	1
Rio Branco	AC	N	Eficiente	1	1	Constante	1	1	1
Macapá	AP	N	Eficiente	1	1	Constante	1	1	1
Palmas	TO	N	Eficiente	1	0,48	Decrescente	1	1	1
São Luís	MA	NE	Eficiente	1	0,61	Decrescente	1	1	1
Teresina	PI	NE	Eficiente	1	0,57	Decrescente	1	1	1
Maceió	AL	NE	Eficiente	1	1	Constante	1	1	1
Salvador	BA	NE	Eficiente	1	1	Constante	1	1	0,99
Belo Horizonte	MG	SE	Eficiente	1	0,36	Decrescente	1	1	1
Vitória	ES	SE	Eficiente	1	1	Constante	1	1	1
Curitiba	PR	S	Eficiente	1	0,59	Decrescente	1	1	1
Florianópolis	SC	S	Eficiente	1	1	Constante	1	1	1
Cuiabá	MT	CO	Eficiente	1	0,48	Decrescente	1	1	0,8
Goiânia	GO	CO	Eficiente	1	0,61	Decrescente	1	1	0,94
Brasília	DF	CO	Eficiente	1	1	Constante	1	1	1
João Pessoa	PB	NE	Ineficiência Fraca	0,98	0,65	Decrescente	1	0,87	1
Manaus	AM	N	Ineficiência Fraca	0,97	0,94	Decrescente	1	1	1
Belém	PA	N	Ineficiência Fraca	0,94	0,66	Decrescente	0,85	1	0,88
Boa Vista	RR	N	Ineficiência Fraca	0,94	0,74	Decrescente	1	1	1
Recife	PE	NE	Ineficiência Fraca	0,91	0,58	Decrescente	0,84	1	1
São Paulo	SP	SE	Ineficiência Fraca	0,91	0,48	Decrescente	0,9	0,92	0,99
Aracaju	SE	NE	Ineficiência Fraca	0,91	0,69	Decrescente	0,92	1	1
Porto Alegre	RS	S	Ineficiência Fraca	0,89	0,76	Decrescente	0,84	0,97	0,94
Natal	RN	NE	Ineficiência Fraca	0,87	0,73	Decrescente	0,9	0,95	0,84
Rio de Janeiro	RJ	SE	Ineficiência Fraca	0,85	0,68	Decrescente	0,83	0,79	0,87
Fortaleza	CE	NE	Ineficiência Fraca	0,84	0,62	Decrescente	0,89	0,94	0,86
Campo Grande	MS	CO	Ineficiência Fraca	0,81	0,39	Decrescente	0,90	1	0,95
			Média (n = 27)	0,96			0,96	0,98	0,97
			Média AD (n = 23)	0,99			0,98	0,99	0,98
			Média OS/FEDP (n = 4)	0,87			0,87	0,9	0,91

UF: Unidade Federada; E_Técnica_: eficiência obtida por DEA VRS-O; E_Global_: eficiência obtida por DEA CRS-O; AD: administração direta; OS/FEDP: organizações sociais ou fundação estadual pública de direito privado.

Nota: ao interpretar os escores consideramos: eficiente (1); fraca ineficiência (0,8 a 1); moderada ineficiência (0,6 a 0,8); forte ineficiência (menor que 0,6).

Os retornos de escala dos processos de APS em cada capital no ano de 2019 (Tabela 2) foram calculados a partir da estimação da E_Global_. Dentre as capitais eficientes, oito operam na escala de transformação ideal (constante) ao transformar *inputs* em *outputs*. As outras sete não operam na escala mais produtiva (decrescente) e poderiam obter ainda mais *outputs* (Y_i_). Conforme esperado, todas as capitais ineficientes operam em uma escala não ideal (decrescente) ao produzir *outputs*, ou seja, caso haja um incremento de 10% no gasto *per capita* (X_i_) é esperada menor melhora percentual nos *outputs*.

Os escores de E_técnica_ da Tabela 2 foram estudados, separadamente, para as capitais com unidades da APS sob AT, que apresentaram, em 2019, média de E_técnica_ = 0,87, inferior àquela das capitais que adotam AD de E_técnica_ = 0,99. Essa diferença tem aumentado nos 12 anos.

Na Tabela 2 também são apresentadas as análises transversais de E_Técnica_ para os anos 2008, 2012, 2016. Doze capitais em AD se mantiveram eficientes em todos os anos, ao passo que os municípios em AT apresentaram, consistentemente, ineficiência fraca em APS, ou seja, no período estudado a relação entre investimentos na APS e resultados obtidos é inferior àquela das capitais eficientes.

A AED fornece ainda os valores dos indicadores na estimativa de metas para os *outputs* (Y_i_) das capitais não eficientes (Tabela 3). Exemplificando, São Paulo precisaria, para ser eficiente, simultaneamente: aumentar 6,1% a cobertura em AB e 7,2% a proporção de cura da tuberculose; e reduzir o ICSAB (1,2), a taxa de mortalidade infantil (1,0), a taxa de mortalidade prematura (164,9), a taxa de mortalidade materna (13,5) e a taxa de incidência de sífilis congênita (3,8). Essas metas poderiam auxiliar a busca por maior eficiência.

**Tabela 3:** Valores dos indicadores e metas, por capital não eficiente, em 2019.

Capital	C_AB		ICSAB		Tx_M_Inf		P_Cura_Tb		T_M_Prem		T_M_Mat		TX_SIFILIS	
	A	M	A	M	A	M	A	M	A	M	A	M	A	M
João Pessoa	92,9	94,7	14,8	14,1	12,7	9,4	66,8	68,1	721,8	548,7	57,9	45,5	9,4	5,1
Manaus	46,3	69,1	24,0	14,0	13,8	13,4	69,9	77,8	524,2	509,5	68,5	47,5	16,0	5,1
Belém	39,4	51,2	21,9	10,2	15,5	13,6	70,2	74,6	649,1	558,6	69,9	46,2	6,4	5,1
Boa Vista	61,1	66,8	28,0	14,4	14,2	12,1	71,4	76,3	530,3	496,2	56,0	52,4	4,6	4,3
Recife	64,8	70,9	14,3	12,9	11,5	10,5	64,9	71,0	811,3	543,3	28,4	26,0	26,9	4,5
São Paulo	62,6	68,7	13,3	12,1	11,2	10,2	73,8	81	739,8	574,9	62,4	48,9	7,5	3,7
Aracaju	70,7	78,0	14,4	13,1	16,9	14,2	66,8	73,7	579,3	525,6	46,4	42,1	16,4	5,9
Porto Alegre	67,9	75,9	17,1	13,2	8,7	7,8	52,6	58,7	928,5	644,5	24,2	21,7	25,4	6,5
Natal	52,5	60,6	13,0	11,3	12,9	10,5	57,8	68,6	706,2	612,5	37,6	32,6	26,5	6,2
Rio de Janeiro	54,9	64,9	13,6	11,5	12,2	10,3	65,0	77,0	926,4	585,4	80,7	47,1	14,8	4,3
Fortaleza	44,2	60,7	16,9	14,2	11,7	9,8	60,3	71,6	617,9	520,2	40,1	33,7	16,0	4,7
Campo Grande	48,4	64,7	14,8	12,1	9,3	7,5	51,1	62,8	706,5	575,5	49,9	22,5	8,5	5,4

A: valores atuais obtidos; M: meta a ser alcançada; C_AB: cobertura populacional estimada pelas equipes de atenção básica; ICSAB: proporção internação por condições sensíveis à atenção básica; Tx_M_Inf: taxa de mortalidade infantil; P_Cura_Tb: proporção de cura em casos novos de tuberculose pulmonar; Tx_M_Prem: taxa de mortalidade prematura (< 70 anos) pelo conjunto das quatro principais doenças crônicas não transmissíveis; T_M_Mat: taxa de mortalidade materna; T_Sif: taxa de incidência de casos sífilis congênita.

Os resultados das mudanças de produtividade pela comparação do IM e seus componentes (E_IM_, T_IM_), em perspectiva dinâmica da mudança entre fronteiras de eficiência sucessivas (2008 e 2019) foram agrupados segundo os modelos de gestão (AD e AT) (Tabela 4).

**Tabela 4:** Mudanças do Índice Malmquist e de seus componentes entre 2008 e 2019, por agrupamentos de capitais segundo modelo de gestão.

Estatística	Geral			Administração direta			Administração terceirizada		
	IM	E_IM_	T_IM_	IM	E_IM_	T_IM_	IM	E_IM_	T_IM_
Média geométrica	0,70	1,02	0,69	0,66	1,04	0,64	0,94	0,90	1,04
Média	0,84	1,05	0,80	0,82	1,07	0,76	0,97	0,93	1,05
Coeficiente variação	45,65	25,34	39,69	49,00	25,02	42,93	23,04	23,23	4,05
Mínimo	0,09	0,58	0,09	0,09	0,58	0,09	0,65	0,66	0,99
Máximo	1,59	1,61	1,15	1,59	1,61	1,15	1,27	1,25	1,10

Nota: para o cálculo dos valores os Y_i_ de 2008 foram corrigidos pelo Índice Nacional de Preços ao Consumidor Amplo para 2019.

IM: Índice Malmquist (IM) e seus componentes: Eficiência (EIM) e Tecnologia (TIM).

Houve redução da produtividade setorial (Geral), com $\mathrm{IM}=0,70\left(0,69^{*}1,02\right)$ que decorreu da involução (< 1) tecnológica (T_IM_ = 0,69) e da mudança positiva (> 1), muito pequena, na eficiência (E_IM_ = 1,02). Como efeito, em média, a APS involuiu (IM) na conversão de *inputs* (X_i_) em *outputs* (Y_i_). A evolução de E_IM_ expressa o uso da escala ideal (adequação ao porte mais produtivo) e o ganho de eficiência técnica ao converter insumos em produtos (X_i_ utilizado em 2008 gerou menos Y_i_ em 2019).

Houve também involução média do IM em ambas as formas de gestão da APS. A APS sob AD evoluiu a Eficiência (E_IM_) e involuiu em tecnologia (T_IM_) enquanto sob AT involuiu a Eficiência (E_IM_) e evoluiu em tecnologia (T_IM_). A mudança negativa nos escores T_IM_ não resulta da observação empírica da tecnologia nas capitais, mas da comparação entre os seus afastamentos à fronteira de eficiência AED VRS−O, medidos nos tempos t (2008) e t+1 (2019). A menor variação (CV, max, min) nos escores sob AT, indicam cidades mais homogêneas na oferta de APS.

## DISCUSSÃO

A análise da eficiência da APS nas capitais brasileiras no período 2008–2019 resultou em 15 capitais com AD eficientes, ao passo que outras 12 apresentaram ineficiência de fraca intensidade. Entre as ineficientes figuram as capitais com administração cedida a terceiros (AT).

Há predomínio da AT no Rio de Janeiro, Porto Alegre, São Paulo e Fortaleza, o que inclui as duas capitais mais populosas. Esse resultado se alinha com levantamento anterior^19^, que indicou predominar estabelecimentos municipais de saúde administrados por terceiros na Região Sudeste (72,4%), seguido da Sul (15,2%) e Nordeste (8,0%). Destacam-se as Fundações no Sul e as OS no Sudeste, que representam 83,3% das instituições terceirizadas.

A gestão em saúde por OS tem presença expressiva nas cidades ou estados de maior poder econômico, com relevância para a sua origem e maior número no estado de São Paulo^20^, ratificando seu caráter empresarial e localização nos principais polos econômicos do país. Outro estudo^21^ corrobora esse achado ao demonstrar a expansão geográfica das dez maiores OS do país, presentes em 17 estados, com concentração no eixo sul-sudeste, de maior dinamismo econômico. Da mesma forma, as capitais com maior percentual de cobertura por planos de saúde estão nas sete capitais dessas regiões.

Todas as capitais investem, ou superam, o percentual mínimo de aplicação de recursos em saúde, o que é consistente com outros autores. Desde 2004, todos os municípios brasileiros superam o percentual mínimo (15%), com média de 22,5% em 2017^22^.

O limite legal de gastos com pessoal no poder executivo municipal (54%) determinados pela LRF, é um dos principais argumentos para a implantação de AT^23^, mormente na APS. No Rio de Janeiro, a terceirização não reduziu o percentual de 56,2%. Também é apontada^23^ redução do esforço fiscal no Rio, que aplicou 25,5% de receita própria em 2016, com posterior redução iniciada, em 2017, quando investiu apenas R$ 3,31 *per capita*/dia em saúde, valor abaixo da média nacional (R$ 3,38 *per capita*/dia)^24^.

Os maiores gastos *per capita* em APS, em 2020, foram realizados em Florianópolis (R$ 517,22) e São Paulo (R$ 403,64), o que pode sugerir baixa influência do modelo de gestão na priorização da APS nos gastos em saúde. Todavia, a cobertura populacional na APS em Florianópolis é historicamente superior à de São Paulo capital^5^.

Ao longo do tempo, houve estabilidade nos escores de eficiência para 12 das 15 capitais sob AD. Essa regularidade está em sintonia com um estudo que indicou Florianópolis, Belo Horizonte e Curitiba como referências nacionais de forte rede de APS com ênfase na ESF^25^. Similarmente, São Paulo não ser eficiente em todos os anos estudados reafirma análise realizada em 2015^26^, de que a experiência de administração gerencial pelo setor privado na capital paulista não alcança maior eficiência mediante o combate ao patrimonialismo.

Os escores de E_Técnica_ das capitais (Tabela 2) foram obtidos em diferentes condições de investimento em saúde. Considerados valores corrigidos para 2019, a APS sob AD reduziu o gasto médio de R$ 232,86/hab para R$ 153,08/hab entre 2008 e 2019 (-52,1%), enquanto as capitais com AT aumentaram de R$ 127,00/hab para R$ 211,94/hab (+66,9%). Há desproporção de investimentos relacionada às escalas de produção decrescentes e ineficiências das capitais sob AT, que têm maior gasto *per capita* (X_i_) sem a correspondente melhora da condição de saúde (Y_i_). Reitera-se que a busca de eficiência pela APS pública é limitada pela continuada asfixia no financiamento^9,17^.

A análise da mudança de produtividade (IM e seus componentes E_IM_, T_IM_), entre 2008 e 2019, também evidenciou diferenças entre modelos de gestão da APS. Ambos apresentaram involução na produtividade média (IM). Nas capitais sob AD esse efeito foi maior pelo recuo tecnológico (T_IM_) e simultâneo incremento de eficiência ao gerar resultados (E_IM_ = 1,04), pela melhora na eficiência técnica pura e de escala. Inversamente, nas capitais sob AT (Fortaleza, Rio de Janeiro, São Paulo e Porto Alegre) houve evolução tecnológica (T_IM_ = 1,04), relacionada à capacidade de adequação das capitais à tecnologia disponível, porém com redução de eficiência. Esses resultados são consistentes com a redução dos investimentos e também podem decorrer da maior heterogeneidade entre as capitais sob AD.

Vale considerar que a eficiência produtiva pode não ser efetiva em atender demandas de saúde^9^. Essa questão é importante no contexto das avaliações dos processos de pactuação entre entes federados, em que a contagem do número de indicadores com metas atendidas pelos municípios pode permitir a sua comparação, mas não a suposição de sua eficiência.

Nossos resultados estão alinhados com estudos realizados em outros contextos. Melhor prestação em hospitais filantrópicos sobre os privados com fins lucrativos^3^; ausência de comprovação de superioridade de modelos alternativos à AD, sendo ainda escassas as evidências sobre impactos positivos da gestão em arranjos público-privados^27,28^. Ademais, a privatização do Sistema Único de Saúde (SUS) como solução para maior eficiência, tem sido tratada como mito^9^.

A revisão de literatura internacional também não encontrou evidências de maior eficiência em prestação pública ou privada no setor saúde. O aumento da privatização do NHS mediante subcontratação de serviços a empresas com fins lucrativos correspondeu à queda de qualidade na assistência em saúde, com aumento de mortalidade evitável^29^, assim como houve piora dos cuidados e resultados em saúde em outros países de renda elevada^30^.

As limitações deste estudo podem decorrer do método e dos dados utilizados. A opção pelo modelo adotado (AED VRS−O e índice Malmquist) produziu resultados consistentes, entretanto destacamos que suas fronteiras de eficiência (vertical e horizontal) podem gerar eficiências que não atendem perfeitamente ao conceito de Pareto-Koopmans. Teria sido possível utilizar modelos mais flexíveis e dinâmicos de AED, mas eles não são isentos de limitações intrínsecas, em especial as suposições de aditividade, divisibilidade, proporcionalidade e certeza. Quanto às variáveis, ressaltamos que a quantidade de DMU não permitiu o uso de formas estocásticas de AED. Quanto aos dados, os indicadores utilizados podem influenciar a eficiência estimada pela incompletude ao representar a APS e, ainda, apesar de haver uma relação lógica consistente entre insumos e produtos utilizados, a eficiência pode ser influenciada por fatores socioeconômicos concorrentes e exógenos à APS, que não foram estudados.

## CONCLUSÕES

O alcance da eficiência não representa, necessariamente, satisfação de necessidades de saúde. Os resultados indicam que unidades da APS sob AD são mais eficientes do que as sob AT e não apontam evidências de ganho de eficiência na APS das capitais brasileiras pela adoção de modelos alternativos de gestão. Isso confronta os benefícios disseminados pelo pensamento neoliberal e incorporados pela nova gestão pública e, ainda, contrasta com a suposição de existir maior eficiência da prestação de serviços públicos com a adoção de fundamentos do mercado e inclusão de instituições privadas para qualificar políticas sociais.
